# Systematic comparison of methods for offline breath sampling

**DOI:** 10.1007/s00216-025-06025-5

**Published:** 2025-08-05

**Authors:** Mark Woollam, Andrew Christensen, Eray Schulz, Serenidy Eckerle, Michael D. Davis, Don B. Sanders, Mangilal Agarwal

**Affiliations:** 1 Department of Chemistry & Chemical Biology, Indiana University Indianapolis, Indianapolis, IN 46202 USA; 2 Integrated Nanosystems Development Institute, Indiana University Indianapolis, Indianapolis, IN 46202 USA; 3 Division of Pulmonology, Allergy, and Sleep Medicine, Riley Hospital for Children at Indiana University School of Medicine, Indianapolis, IN 46202 USA; 4 Wells Center for Pediatric Research, Riley Hospital for Children at Indiana University School of Medicine, Indianapolis, IN 46202 USA; 5 Department of Biomedical Engineering & Informatics, Indiana University Indianapolis, Indianapolis, IN 46202 USA

**Keywords:** Exhaled breath, Volatile organic compounds (VOCs), Gas chromatography-mass spectrometry (GC–MS), Thermal desorption, Method standardization

## Abstract

Harnessing the potential of exhaled breath analysis is an emerging frontier in medical diagnostics, given breath is a rich source of volatile organic compound (VOC) biomarkers for different medical conditions. A current downfall in this field, however, is the lack of standardized and widely available methods for offline sampling of exhaled VOCs. Herein, strides are taken toward the standardization of breath sampling in Tedlar bags by exploring several factors that can impact VOC heterogeneity, including tubing material, chemical composition of collection bags, breath fractionation, exhalation volume, and transfer flow rate. After bag-based sampling standardization, performance was benchmarked using two offline breath sampling methods, Tedlar bags and the Respiration Collector for In Vitro Analysis (ReCIVA). Three volunteers from the laboratory with no known respiratory diseases donated ≥ *n* = 5 samples collected onto adsorption tubes via each method, which were analyzed through thermal desorption (TD) coupled with gas chromatography-mass spectrometry (GC–MS). Data processing revealed a set of 15 highly reliable on-breath VOCs detected across volunteers, and most analytes (except indole) demonstrated higher sensitivity using Tedlar bags. Calculating relative standard deviation (RSD) values showed Tedlar bags were also significantly more reproducible compared to the ReCIVA (*p* < 0.03). Agreement between the two methods was demonstrated through correlating VOC signals with high statistical significance (*R*^2^ = 0.70), indicating both devices are well situated for biomarker discovery applications.

**Graphical Abstract:**

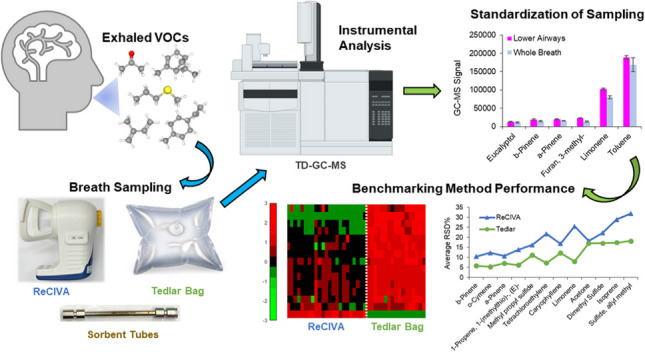

**Supplementary Information:**

The online version contains supplementary material available at 10.1007/s00216-025-06025-5.

## Introduction

Volatile organic compounds (VOCs) in exhaled breath, and other non-invasive biological fluids including urine and sweat, are potentially useful biomarkers for diagnosing an array of medical conditions [1, 2]. Although viable in an array of sample types, exhaled breath is of significant interest considering it is highly non-invasive, practically limitless in supply, and most importantly, a rich source of VOCs for biomarker discovery [3]. The biochemical rationale for harnessing the potential of VOCs as biomarkers is that endogenous volatile molecular components are byproducts of metabolic pathways which can be manipulated uniquely by different human diseases [4, 5]. For example, acetone and other ketone bodies are well-established biomarkers for diabetes [6, 7], since they are biosynthesized in the liver through the oxidation of polyunsaturated fatty acids (PUFAs). Once glycogen is significantly reduced, the ketones can be introduced into the blood where they can be distributed throughout the body and undergo alveolar exchange to ultimately be expressed in breath. Beyond acetone, numerous VOCs in exhaled breath have been implicated as biomarkers for other medical conditions including but not limited to different types of cancer [8–13], liver disease [14–17], as well as infectious diseases (bacterial and viral) [18, 19].

The gold standard for analyzing VOCs in exhaled breath is gas chromatography-mass spectrometry (GC–MS) because it can chromatographically separate, quantify, and structurally elucidate volatile analytes within a complex sample matrix [20]. There are also well-established methods for preconcentrating and capturing VOCs in breath, which include solid phase microextraction (SPME) and thermal desorption (TD) using adsorption tubes. For example, recent literature has demonstrated that glass sampling bottles can be coupled with SPME for different breath-based applications [21, 22]. In general, SPME fibers are ideal when the sample is in the aqueous phase (sweat, urine, blood) with limited volume, where adsorption tubes are more ideal for collecting large quantities of samples in the gas phase [23]. Therefore, adsorption tubes are the optimal method for capturing VOCs in breath, but it should be noted that an additional TD unit is required for GC–MS analysis. Although robust methods for instrumental analysis are straightforward and widely available, those for offline breath sampling are not. To date, there is no widely accepted or standardized method for collecting VOCs in exhaled breath [24]. This is important given there are several factors that could potentially introduce intra- or inter-method heterogeneity.

One important factor to consider is that exhaled breath is comprised of multiple fractions including dead space, mixed air, and alveolar (end-tidal phase) breath. Different phases have varying VOC compositions, and the end-tidal fraction has been shown to contain the most rich and reproducible profile [25, 26]. The end-tidal phase can be isolated from other portions through monitoring exhaled CO_2_ using capnography where exhaled breath is only collected once the alveolar plateau is achieved. Additionally, exhaled volume and other respiratory factors including inhalation/exhalation rate as well as hypo/hyperventilation conditions can impact VOC sensitivity and reproducibility [27, 28]. Beyond aspects of respiratory physiology, there are different collection materials and devices used to sample exhaled breath. The simplest and most widely utilized devices for breath sampling are polymer-based collection bags [24, 29], where VOCs can be transferred to adsorbent tubes using a flow-regulated pump. However, drawbacks to bag-based techniques include the fact that fractionation is not inherently achieved, Tedlar collection bags can contain background VOCs which may interfere with analysis, and VOCs must be transferred immediately to sorbent tubes to avoid losses in sensitivity [30, 31]. For these reasons, there have been efforts to develop more direct methods for exhaled VOC collection onto adsorption tubes.

The Respiration Collector for In Vitro Analysis (ReCIVA) developed by Owlstone Medical is a state-of-the-art system designed to more directly collect different fractions of breath onto sorption tubes [32, 33]. Briefly, this system uses flow/CO_2_ sensors to focus analysis on a particular fraction of breath (typically lower airways), where automated pumps draw VOCs onto up to four adsorption tubes simultaneously at a consistent flow rate. Although the ReCIVA represents a significant advancement in breath-based technology, it is costly, and its performance has not been widely qualified for analysis of VOCs. Previous studies have evaluated the ReCIVA device [34–36], but most of these studies focus on disease-specific applications and do not benchmark method performance or compare results using complementary techniques. A previous study published by Harshman et al. qualified and compared the performance of two different but identical ReCIVA systems. Analyses revealed that breathing rate into the device did not impact the observed VOC data, and that the results from the two different systems mirrored each other [37]. More recently, Gilio et al. compared the performance of three different breath sampling methods, including Tedlar bags, the ReCIVA, and a newly developed system. Through analysis of breath samples collected from 10 healthy volunteers, it was determined that higher VOC levels were quantified using the Tedlar bags relative to the ReCIVA [38]. Herein, the authors take a deep dive into two well-known methods for offline breath sampling of VOCs (Tedlar bags and the ReCIVA), through exploring quantitative/qualitative factors that affect the fidelity of breath-based data. The overarching purpose of this study is to evaluate and contrast the effect of sampling factors, such as sampled breath volume, fractionation, material chemistry, and gas dynamics (flow and transfer volume), on the quality of data recovered from breath samples.

## Materials and methods

### Materials and instrumentation

Three-liter Tedlar and Multi-Layer Foil gas sampling bags were purchased from Restek (Bellefonte, PA, USA). ViroMax viral and bacterial filters (A-M Systems; Sequim, WA, USA) were used to remove any viral or bacterial agents from bag-based breath samples. A Philips NM3 capnograph (Murrysville, PA, USA) was used to monitor CO_2_ levels and fractionate breath when collecting into Tedlar bags. Perfluoroalkoxy (PFA), Tygon, and polytetrafluoroethylene (PTFE) tubing with various inner diameters (3/16-in., 1/4-in.) were obtained from Saint-Gobain (Courbevoie, France), Cole-Parmer (Vernon Hills, IL, USA), or Chemglass Life Sciences (Vineland, NJ, USA). An MCS-series mass flow controller purchased from Alicat Scientific (Marana, AZ, USA) was used to transfer VOCs collected in bags onto adsorption tubes for TD-GC–MS analysis. A ReCIVA system (serial no. 1240) equipped with a CASPER Portable Air Supply was also used for breath sampling and acquired from Owsltone Medical (Milton, Cambridge, UK). Using both collection devices, VOCs were captured using bio-monitoring-stainless steel adsorption tubes (C2-AAXX-5149) with analytes thermally desorbed using a Centri 180 system manufactured by Markes International (Bridgend, UK). A 7890 A GC coupled with a 7200-quadrupole time-of-flight (QTOF) MS manufactured by Agilent (Santa Clara, CA, USA) was used to analyze VOCs. A Restek Rxi-5 ms GC column 30 m in length, 0.25 mm in internal diameter, and with a 0.25-μm film thickness was used to chromatographically separate exhaled breath VOCs.

### Volunteer selection and sampling

The study has two main series of experiments: (1) optimization of bag-based sampling parameters and (2) comparison/benchmarking the performance of breath sampling platforms. An experimental overview of parameters for both series is given in Appendix Table A1. To explore and optimize different variables involved in sampling breath using Tedlar bags, breath was analyzed from a single volunteer from the laboratory. Furthermore, experiments and associated data interpretations were consolidated to a single day, and conditions were interpolated (in sample collection and instrumental analysis) to avoid undesirable VOC variation. For each parameter optimized, at least *n* = 3 samples were collected for each condition. After Tedlar bag optimization, a total of three volunteers provided exhaled breath samples using both methods (ReCIVA and Tedlar bag), each on a different day. Laboratory volunteers were male and between the ages of 25 and 30 with no respiratory or other medical symptoms. All lab participants avoided food/drinks (other than water), smoking, and brushing their teeth at least 1 h before the scheduled breath collection events. Each breath sample was taken sequentially approximately every 30 min, with the goal of keeping a “constant” breath profile. Prior to breath sampling, background samples were collected using the CASPER Portable Air Supply on both devices (*n* = 3 for Tedlar bags, *n* = 4 for ReCIVA). For each of the volunteers, at least *n* = 5 tubes were collected on a single day using both the ReCIVA and Tedlar bags. It should be noted that the Tedlar bag method required the collection of more background/breath samples, as one bag was used for each adsorption tube analyzed (four tubes can be collected using one sample on the ReCIVA). Immediately after collection (or transfer), the adsorption tubes were capped and analyzed accordingly through TD-GC–MS. All the laboratory volunteers were consented, and all study procedures abided by the Indiana University Institutional Review Board protocol (IRB # 15542).

### Tedlar bag breath sampling and optimization

Prior to breath sampling, brand new Tedlar bags were equilibrated with nitrogen for at least 3 min using a previously published procedure [39–41]. To collect VOCs into a Tedlar bag, healthy volunteers from the laboratory were instructed to breathe tidally through a viral filter interfaced with a capnograph. These components were connected to tubing that delivered VOCs to a Y-junction that houses (1) a two-way valve and (2) the collection bag. To capture end-tidal phase breath, the volunteer began breathing with the two-way valve open while the bag valve remained closed; dead space and mixed-air portions were thus vented from the system into the ambient environment. Once the alveolar plateau was determined through capnography, the two-way valve was closed, and the Tedlar bag valve was opened, thus collecting a focused fraction of end-tidal phase breath. Whole breath samples can also be collected using this system through ensuring the two-way valve remains closed and the Tedlar bag valve remains open during the entirety of breath sample collection. For all breath samples, Tedlar bags were filled to approximately 80% capacity, and mass flow controllers were used to regulate VOCs from the bag to an adsorbent tube at an optimized volume and flow rate. It should also be noted that all samples were transferred immediately after collection to avoid any VOC loss. Prior to benchmarking method performance, different qualitative/quantitative factors were explored and optimized. Qualitative factors that were explored include the use of Multi-Layer Foil/Tedlar bags, the chemical composition of tubing used for collection/transport, and exhaled breath fractionation (whole vs. lower airway breath). Quantitative variables of interest included the volume and flow rate used to transfer VOCs from Tedlar bags into adsorption tubes. Experiments were also undertaken to understand the effects of increasing/decreasing dry purge rates/volumes.

### ReCIVA exhaled breath sampling

Breath sampling methods using the ReCIVA have been previously optimized and published by researchers at Owlstone Medical [33], and therefore were adopted for the current analyses. Briefly, a silicone face mask was utilized with the ReCIVA to collect exhaled breath samples in this study. Although the device is also available for operation with a mouthpiece that decreases the signal of background VOCs, the mouthpiece itself is not operational for previous generations of the ReCIVA and may not be applicable in different populations including children or those with respiratory complications. Using the ReCIVA, 1.25 L of lower airway (fractionated) breath was collected using two pumps (right and left) across four adsorbent tubes at a flow rate of 200 mL/min. It should be noted that some samples were collected onto two tubes and others were collected onto four, given operational difficulties regarding the right pump over the course of experiments. As breath is collected into the ReCIVA, filtered ambient air is provided for inhalation through use of the CASPER Portable Air Supply which implements an activated carbon filter to remove contaminants from the environment. Background samples were collected using the same parameters as breath via the ReCIVA, but with the sampling port blocked and pumps operating continuously.

### Instrumental analysis using TD-GC–MS

Prior to collection, all adsorption tubes were thermally conditioned to remove any contaminant VOCs for 30 min (split flow rate = 50 mL/min). Once VOCs were collected into adsorption tubes, they were dry purged at a flow rate of 125 mL/min for a total of 9 min. VOCs within the adsorption tube were thermally desorbed onto a trap for 10 min at 270 °C. The flow path temperature was maintained at 150 °C with a standby split flow rate = 10 mL/min. Analytes were desorbed from the trap for 2 min using the maximum heating rate with purge flow = 50 mL/min, and high trap temperature = 300 °C. Once VOCs exited the trap, they entered the inlet of the GC–MS for instrumental analysis. The GC protocol involved maintaining the oven temperature for 2 min at 40 °C followed by a ramp to 100 °C at a rate of 8 °C/min, a 15 °C/min ramp to 120 °C, 8 °C/min to 180 °C, 15 °C/min to 200 °C, and a final ramp of 8 °C/min to 260 °C. Ultra-high-pure helium was used as the mobile phase at a flow rate of 1.2 mL/min. The mass analyzer was implemented in full scan mode, ranging from 26 to 400 m/*z*. The ion source operated at 250 °C with an emission current of at least 5 µA.

### Data processing and analysis

For all experiments, Agilent MassHunter was utilized for data acquisition in centroid format. Regarding the Tedlar bag optimization experiments, method performance was assessed through quantifying the total number of VOCs, the total integrated signal, as well as the levels of unique on-breath VOCs. To broadly analyze the number of VOCs as well as the total GC–MS signal from a given sample, MassHunter Qualitative Workflows was used to deconvolute chromatograms/spectra. To analyze signals of individual compounds, MassHunter Navigator was utilized to integrate total ion chromatograms. MassHunter Profinder was also used in some cases for spectral alignment to visualize the trends of individual VOCs. The data from experiments focusing on benchmarking/comparing method performance was also processed using Profinder, where samples were deconvoluted and spectrally aligned using a retention time (RT) tolerance of ± 0.00% + 0.40 min and a minimum dot product between mass spectral vectors equal to 0.40. A matrix containing *m*/*z* base peaks, RTs and integrated signals for each VOC and corresponding sample was generated. To remove zero values from the data matrix, half of the minimum value was added as a constant to each of the signals (including non-zero values). It should be noted that samples were spectrally aligned in batches according to volunteer (day of analysis).

Once spectrally aligned, different data filters were employed to focus on potentially useful VOCs. For each volunteer, compounds that were not present in at least 80% of samples within a given method were removed. Additionally, VOCs that were not statistically significantly enriched in exhaled breath (relative to background air samples) were also removed from the data matrix. A set of 15 on-breath VOCs were detected to be statistically significantly enriched in breath samples compared to background samples in all volunteers in at least one of the methods (Student’s *T*-test with the Bonferroni approach to correct for multiple comparisons). Here, method sensitivity was undertaken through generating hierarchical heatmaps in Matlab and comparing log_2_ fold change (FC) values for the 15 analytes. Reproducibility of the methods was benchmarked through calculating relative standard deviation (RSD) values for each of the volunteers. Additionally, the data for the 15 VOCs was z-scored (autoscaled) according to volunteer/day of analysis, and principal component analysis (PCA) was implemented to visualize methodological differences. PCA was also utilized on data, which was autoscaled according to method, to observe degrees of method agreement. VOC correlations between the two methods were also assessed through averaging log_2_-normalized signals by volunteer and utilizing linear regression to observe if the results converged. In various statistical comparisons, the Student’s *T*-test was employed using a two-tailed approach (when applicable, paired tests were performed). VOC identification in this study was performed through mass spectral matching using the National Institutes of Standards and Technology (NIST) 17 library, as well as by comparing the analyte’s nonpolar retention index (NPRI) in NIST to an experimental NPRI calculated by an instrument-specific calibration curve [42].

## Results and discussion

### Effect of tubing chemical composition

**Fig. 1:**
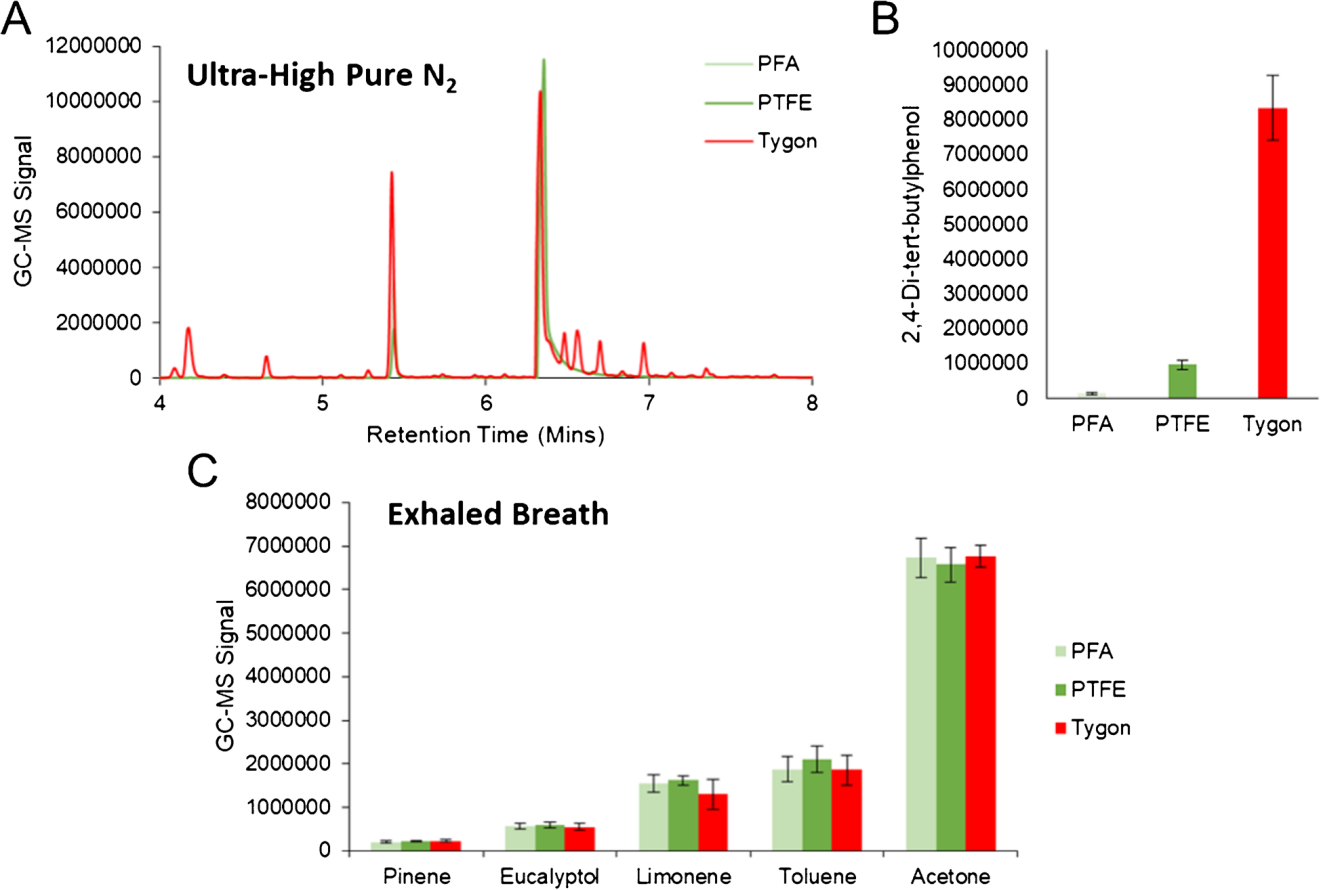
**A** Chromatographic traces of ultra-high pure nitrogen samples collected in Tedlar bags and transferred using tubing of different chemical compositions show that Tygon tubing has a significantly elevated background. **B** Bar plots of 2,4-di-tert-butylphenol in the same samples show elevated levels in Tygon and PTFE tubing. **C** Bar plots show the signal of on-breath VOCs when transferred using the different tubing types, displaying no significant differences

Since sampling through Tedlar bags requires the use of tubing for both collection and transfer to adsorption tube, different tubing chemical compositions were tested (PFA, PTFE, and Tygon). Two different experiments were conducted, one which filled the bag with ultra-high pure nitrogen to elucidate exogenous compounds originating from each tubing type, and another which focused on-breath sampling to ensure efficient transport of exhaled VOCs. It should be noted that for breath collection in these initial experiments, no fractionation was undertaken and therefore did not require the use of any tubing for collection (solely for transfer). In these experiments, 500 mL of gas was transferred from the bag to the adsorption tube at a flow rate of 250 mL/min. Analysis of bags filled with ultra-high pure nitrogen revealed that samples transferred using Tygon tubing contained several unique GC–MS peaks that remained undetected in PFA and PTFE (Fig. 1(A)). Chromatographic peaks at RTs equal to 4.18, 4.66, 6.48, 6.56, 6.70, and 6.97 min were characteristic to Tygon tubing, and displayed base *m*/*z* values equal to 57 or 43. These compounds were comprised of branched/short-chained hydrocarbons and therefore may interfere in the analysis of breath-based VOCs. There was also an array of other saturated hydrocarbons beyond these specific RTs which were characteristic to Tygon tubing. Differences between PFA and PTFE were limited, aside from the fact that 2,4-di-tert-butylphenol was significantly elevated (*p*-value = 0.006) in PTFE relative to PFA (Fig. 1(B)). Regarding experiments analyzing breath, exhaled VOCs across the board did not show any significant differences between the tubing types (Fig. 1(C)), indicating little to no loss among the different chemical compositions. Taken as a whole, it is recommended to utilize PFA as a primary option or PTFE as a secondary alternative in the collection and transport of exhaled VOCs.

### Exploring collection bag material

**Fig. 2:**
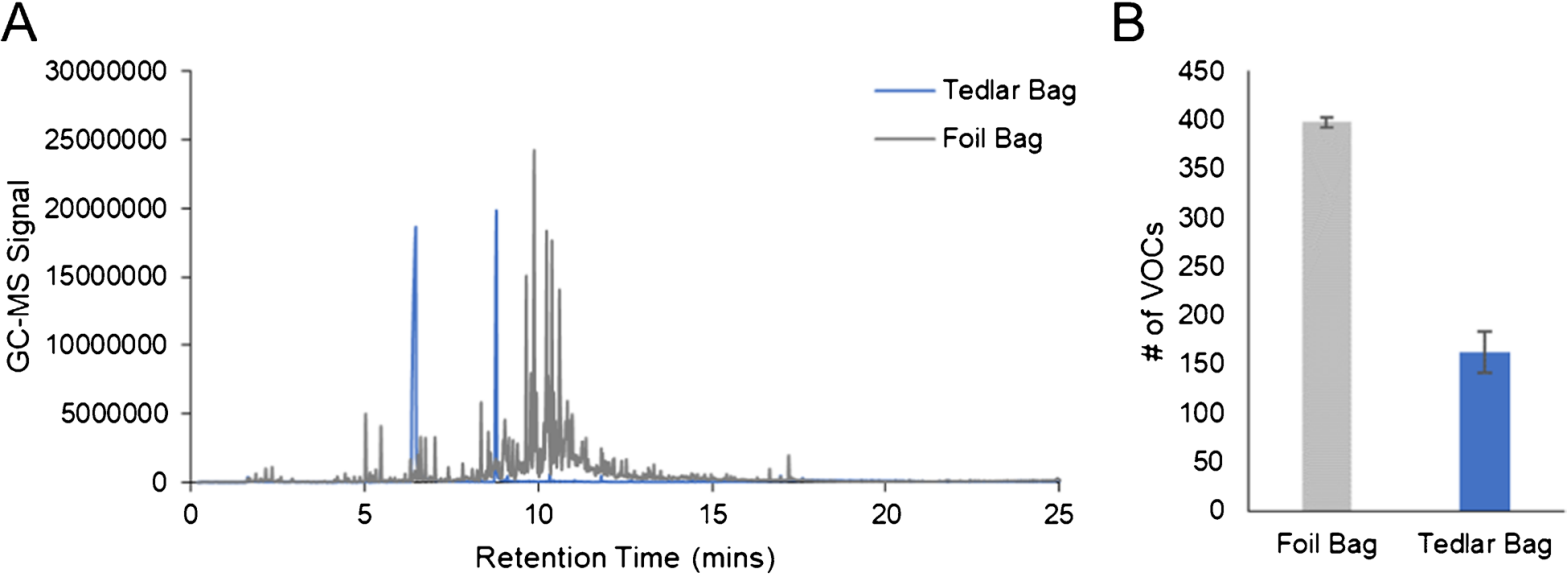
**A** GC–MS chromatograms of ultra-high pure nitrogen samples collected in Multi-Layer Foil and Tedlar bags, along with **B** bar plots illustrating the number of deconvoluted VOCs by sample type, show that Tedlar bags have a significantly reduced background and therefore are optimal for breath collection/analysis

In addition to tubing chemical composition, two different collection bags were tested (Tedlar and Multi-Layer Foil bags) using ultra-high pure nitrogen and the same parameters described above. GC–MS chromatograms for these two sample types are displayed in Fig. 2(A) and show that there are an abundant number of chromatographic peaks with higher intensity in Multi-Layer Foil bags. Aside from N,N-dimethylacetamide (RT = 6.47 min) and phenol (RT = 8.79 min), all other VOC signals observed in the background were lowest in the Tedlar bags. This is further supported by the fact that when all chromatograms were deconvoluted, there was a 244% increase in the number of VOCs detected in the Multi-Layer Foil bag relative to Tedlar (Fig. 2(B)). This difference was statistically significant and reached a *p*-value = 0.001. Based on these results, it was clearly determined that Tedlar bags had a significantly reduced background and therefore would be more ideal for breath sampling compared to Multi-Layer Foil bags. These results align well with general knowledge, as phenol and N,N-dimethylacetamide are known Tedlar bag contaminants [43], and foil bags are better suited for collecting compounds such as methane or hydrogen which would diffuse and be lost in Tedlar bags [44, 45]. Even though these interferents are present in Tedlar bags, this does not impact the analysis for most analytes including those presented in this manuscript. This is because the VOCs of interest are chromatographically resolved with respect to these two chemical interferents (no overlap by RT).

### Exhaled breath fractionation in Tedlar bags

**Fig. 3:**
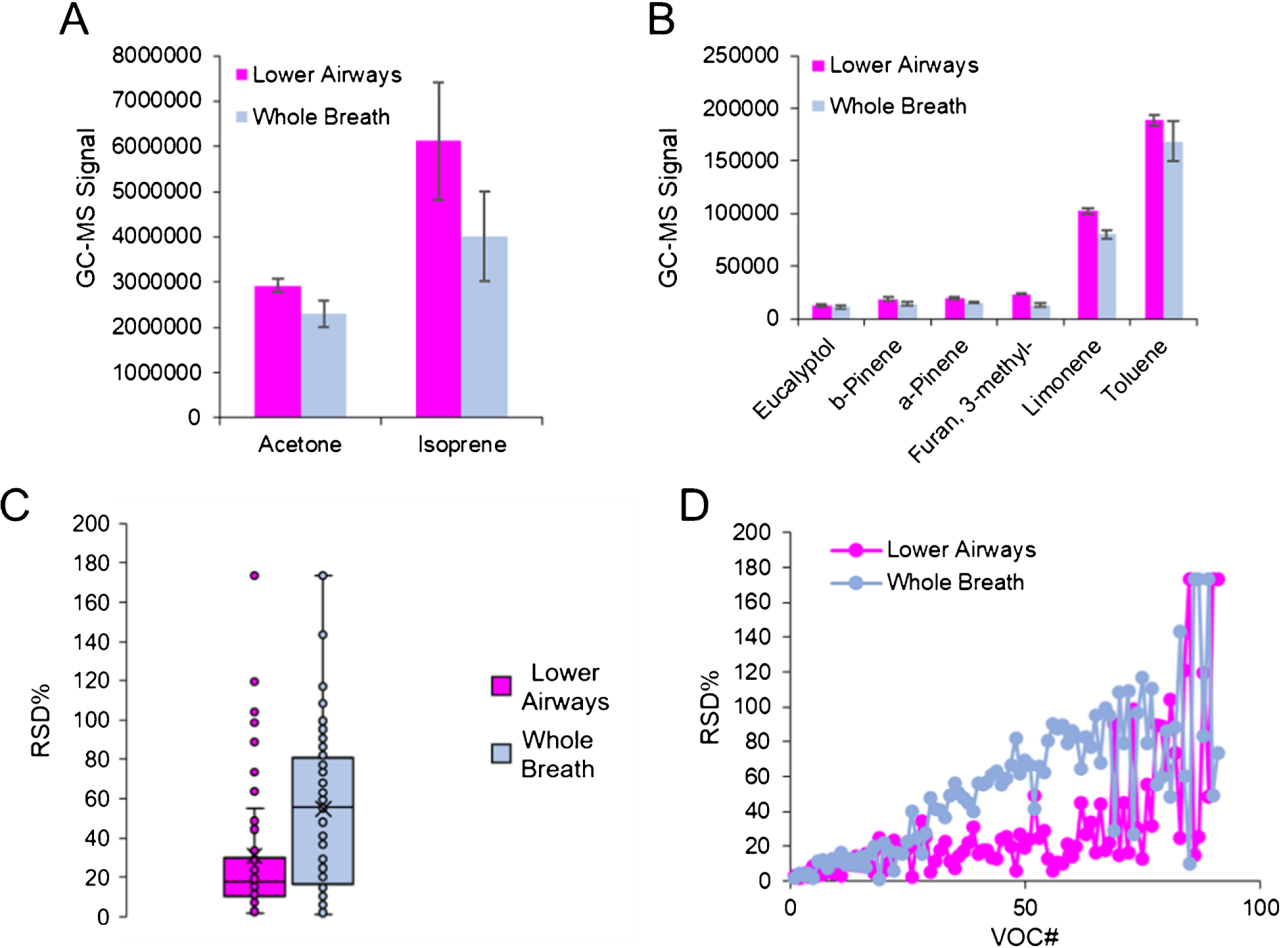
Bar plots for individual VOCs including **A** acetone and isoprene, along with **B** eucalyptol, pinene isomers, and other compounds, show fractionating lower airway breath is more sensitive relative to whole breath. **C** Box/whisker and **D** scatter plots displaying RSD values for untargeted VOCs show a higher degree of reproducibility in fractionated samples

Once the chemical composition of tubing and collection bags was optimized, experiments focusing on the effects of breath fractionation were undertaken. After analyzing whole and lower airway breath samples from a single volunteer, the signals of typical on-breath VOCs were visualized to observe any differences in sensitivity (Fig. 3(A) and (B)). Here, many compounds including acetone, pinene isomers, and limonene demonstrate an elevated signal in lower airway samples compared to whole breath. Other compounds (isoprene, eucalyptol, and more) also trended higher in lower airway breath samples but did not reach statistical significance. It should be noted that on average, the VOCs in breath increased by a factor of 34% when analyzing fractionated breath relative to whole breath. Beyond increases in sensitivity regarding on-breath compounds, other untargeted VOCs were spectrally aligned in samples and reproducibility was assessed (Fig. 3(C) and (D)). Among the 91 VOCs that were filtered based on abundance, there was a significant difference in RSD values where lower airway samples exhibited a higher degree of reproducibility (*p*-value = 0.00001). Taken as a whole, although the increases in sensitivity for fractionating breath were small, they were statistically significant. Moreover, the large differences in VOC reproducibility highlight the necessity to fractionate samples when the goal is to generate the highest quality of breath-based data for biomarker discovery.

### Transfer volume and flow rate

**Fig. 4:**
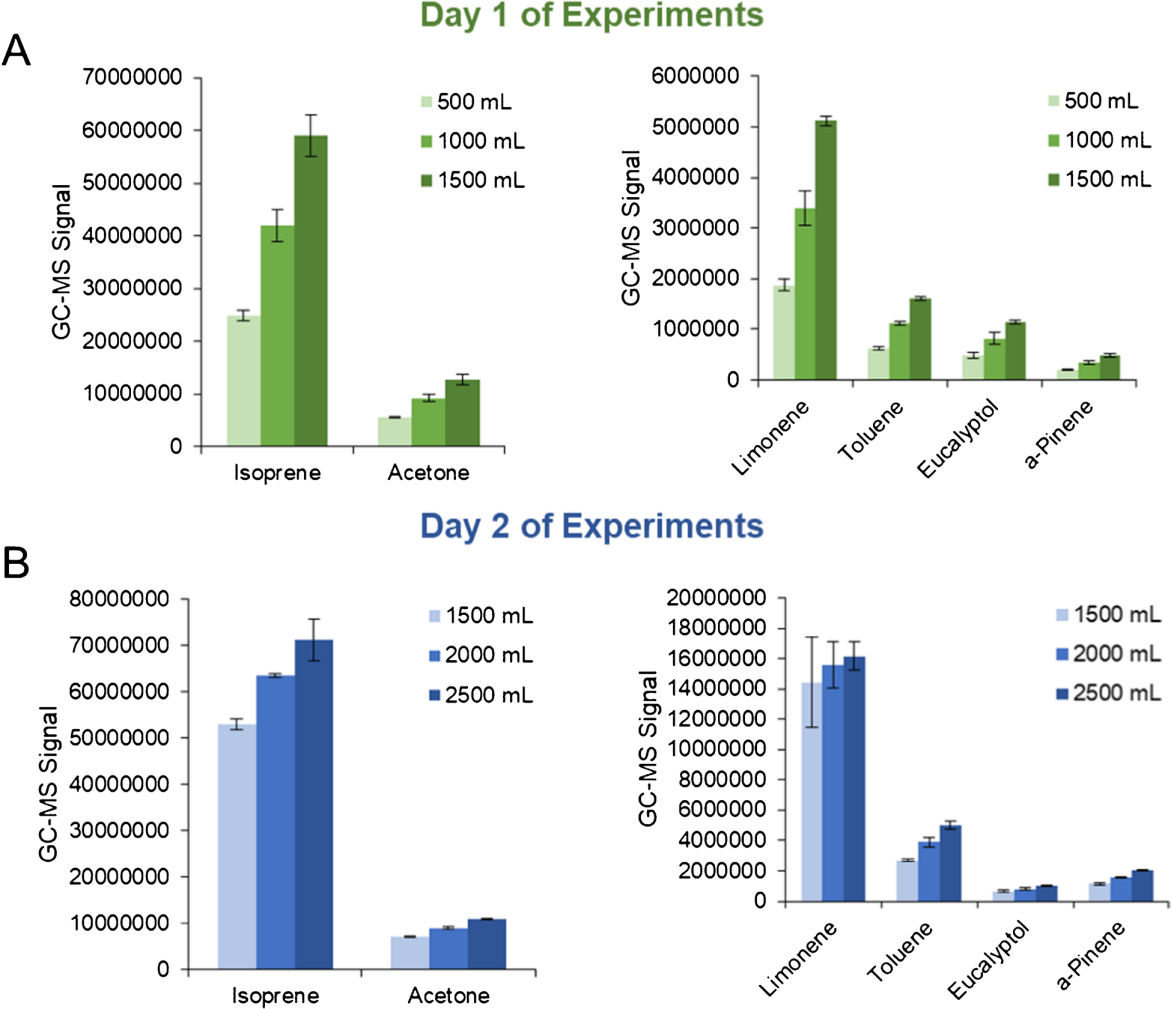
**A** First day of experiments regarding optimization of exhalation volume, showing the signals of on-breath VOCs are positively correlated with volume and 1.5 L is the most optimal condition. **B** The second day of experiments extends analyses to larger volumes, which showed further increases in sensitivity were observed

The volume that is collected/transferred from the bag to the adsorption tube will have a profound impact on the acquired data. Exhalation volume was explored and optimized in this study using one volunteer who provided breath samples on a single day. The first day of experiments, which tested exhalation volumes equal to 500 mL, 1 L, and 1.5 L, showed that the signal of on-breath VOCs, including isoprene, acetone, and limonene, was positively correlated with volume (Fig. 4(A)). These preliminary analyses indicated that 1.5 L was most optimal, and a second day of experiments was undertaken to observe if further increases in sensitivity could be achieved using higher breath volumes. On the second day of analysis, samples were collected from the same volunteer and transferred with volumes equal to 1.5 L, 2 L, and 2.5 L. It should be noted that 1.5 L samples were recollected since it was not assumed that inter-day VOC measurements were comparable. Volumes beyond 2.5 L were not assessed, as this would represent more than 80% of the Tedlar bag’s maximum capacity. The quantitative results are presented in a similar fashion (Fig. 4(B)) and displayed that once again, VOC signals were positively correlated with exhalation volume, and therefore 2.5 L produced superior sensitivity.

**Fig. 5:**
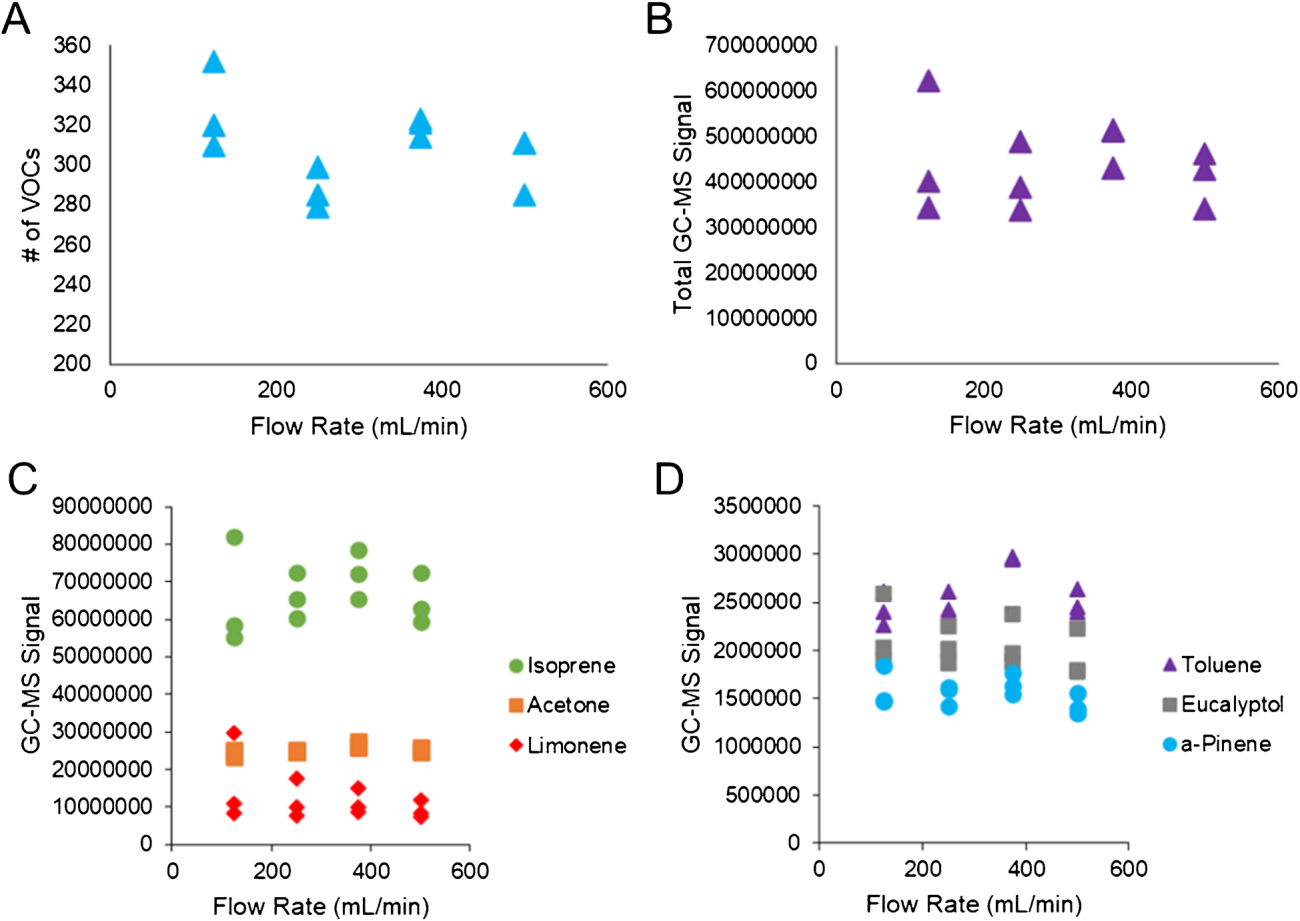
Scatter plots showing **A** number of deconvoluted VOCs and **B** total integrated GC–MS signals as a function of transfer flow rate from the Tedlar bag indicate no correlation or impact. Scatter plots are also produced for **C** acetone, isoprene, limonene, **D** toluene, eucalyptol, and pinene, further illustrating transfer flow rate does not have a quantitative impact on exhaled breath VOC signals

Nonetheless, several factors need to be considered regarding implementation/application. For example, different volunteers will display a range of VOC levels, and for proper quantification, the method needs to detect all signals within the dynamic linear range of the instrument. Increasing the volume of breath analyzed in some cases may elevate the VOC signals out of this linear range, making quantitative analyses difficult or unachievable. Furthermore, because it is challenging to visualize the captured breath volume within a bag, the authors recommend that no more than 2 L of exhaled breath is transferred to an adsorbent tube for subsequent analysis. If target volumes are higher than 2 L, not enough analyte may be transferred onto the tube. It should also be noted that in some biomedical applications, 2 L of breath may decrease chromatographic performance, as well as trapping and ionization efficiency. After exploring exhalation volume, different transfer flow rates were explored in a systematic fashion (125, 250, 375, and 500 mL/min). As shown in Fig. 5(A) and (B), the number of VOCs or the total GC–MS signal was not impacted by varying the transfer flow rate. The integrated signals of key on-breath VOCs were also tabulated (Fig. 5(C) and (D)), which included isoprene, acetone, limonene, and other compounds. These results mirrored those in Fig. 5(A) and (B), further displaying that flow rate has little to no impact on the quantitative results. In summary, when transferring exhaled breath from bags to adsorption tubes, a wide range of flow rates could be employed between 125 and 500 mL/min to generate comprehensive results.

### Assessing the impact of dry purge time and flow rate

A critical part of thermal desorption in breath sampling applications is dry purging. In this process, a stream of nitrogen is passed through the adsorption tube (opposite to the direction of thermal desorption) to remove water from the back of the tube. This allows for a virtually moisture-free desorption of VOCs into the GC–MS, preventing water from interfering with retention times, peak shape, and even resolution. When using a Tedlar bag, a purge method of 5 min at 50 mL/min has been employed per recommendation of Markes International, while Owlstone Medical recommends having a purge time/flow rate of 9 min and 125 mL/min, respectively, for the ReCIVA. More intense dry purging methods are necessary for the ReCIVA, given water vapor condenses inside the adsorption tube during collection. Moisture interference is not as large of a concern in Tedlar bags, given water vapor condenses on the internal surfaces of the bag and therefore does not transfer to the adsorbent tube (to the same degree as the ReCIVA). Therefore, to investigate differences in the dry purge methods, *n* = 3 breath samples for each method were collected in Tedlar bags to observe any VOC losses induced by increasing the purge time/rate. No statistical significance was observed for GC–MS signals between the methods (median *p*-value = 0.40, median RSD = 8.6%). This demonstrates that increasing the dry purge time/rate, which is necessary for analysis by ReCIVA, does not significantly vary VOC results. For the sake of thermal desorption consistency, both methods were run with the 9-min 125 mL/min dry purge method for breath comparisons in this study.

### Comparing sensitivity in ReCIVA and Tedlar bags

After qualitative and quantitative aspects of sampling breath into a Tedlar bag were optimized, background and breath samples were collected from three different volunteers using two methods (Tedlar bags and ReCIVA). Regarding the collection of background samples, *n* = 4 ReCIVA samples and *n* = 3 Tedlar bag samples were sampled/analyzed each day (*n* = 12 and *n* = 9 in total for each method, respectively). For Tedlar bag samples, the first/third volunteer donated *n* = 6 samples and the second volunteer provided *n* = 5 samples (in total, *n* = 17 Tedlar bag breath samples). On the ReCIVA, the first/third volunteer provided *n* = 8 samples, and the second volunteer provided *n* = 7 samples (in total, *n* = 23 ReCIVA breath samples). It should be noted that one tube from the second volunteer was contaminated with saliva (as determined through poor chromatographic performance due to water interference), and therefore was removed from analysis. For each of the methods, 1.25 L of lower airway breath was collected onto adsorption tubes at a flow rate equal to 200 mL/min. It should be noted that although this is not the optimal exhalation volume for Tedlar bags, methods were conformed to previous protocols published by Owlstone Medical regarding ReCIVA operation [33]. Given operation/application of the ReCIVA is best understood by the manufacturer, these parameters were selected for assessment. Once all samples were analyzed instrumentally, the data was spectrally aligned into three batches independently according to the respective day of analysis. After data screening, a total of 15 VOCs were identified to be significantly enriched in exhaled breath samples and were detected across all three volunteers in at least one of the methods. Table 1 lists the identities for each of these analytes, along with their average RT, experimental NPRI, and base *m*/*z* value within the mass spectra.

**Table 1:** List of VOCs significantly enriched in breath and reproducibly detected in at least one method (ReCIVA or Tedlar bag), along with their respective retention time (RT), experimental nonpolar retention index (NPRI), and base *m*/*z* value

#	VOC	RT (avg)	exp. NPRI	Base *m*/*z*
1	Acetone	1.86	567.4874	43
2	Isoprene	1.91	570.5919	67
3	Dimethyl sulfide	1.96	573.6964	62
4	Sulfide, allyl methyl	3.3	656.897	73
5	Methyl propyl sulfide	3.53	671.1777	61
6	1-Propene, 1-(methylthio)-, (E)-	3.87	692.2883	73
7	Tetrachloroethylene	5.2	774.868	166
8	a-Pinene	7.78	935.0602	91
9	b-Pinene	8.1	954.929	93
10	3-carene	9.37	1033.7833	91
11	o-Cymene	9.65	1051.1685	119
12	Limonene	9.74	1056.7566	67
13	Benzothiazole	13.01	1259.7909	135
14	Indole	14.04	1323.7436	117
15	Caryophyllene	16.06	1449.1654	91

**Fig. 6:**
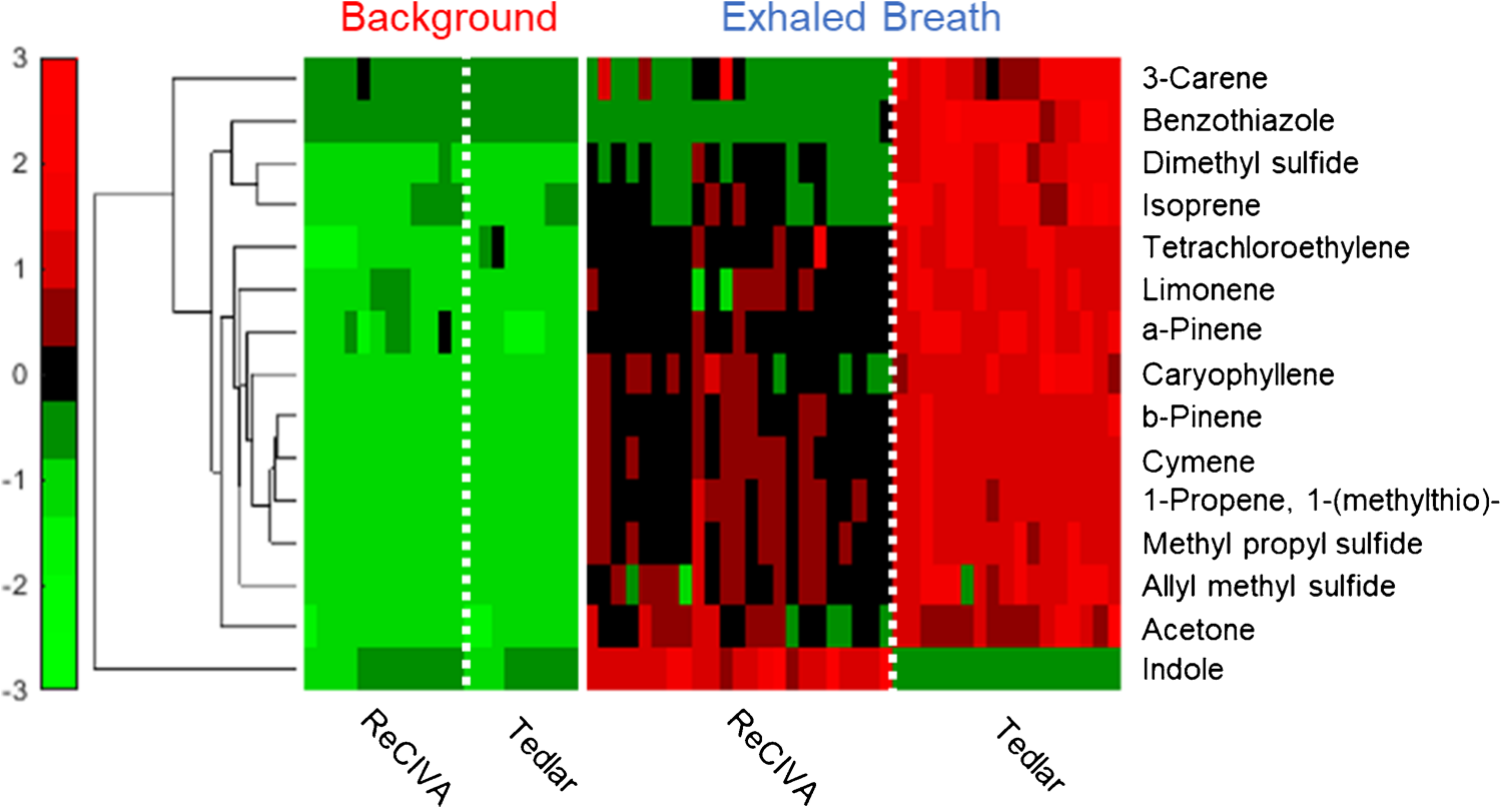
Hierarchical heatmap of the select 15 VOCs in background samples and breath samples collected from three different volunteers shows a more sensitive analysis through use of Tedlar bags relative to the ReCIVA

Although from healthy volunteers, this list comprises several potential VOC biomarkers. For example, limonene has been demonstrated in multiple studies to be a clinically relevant biomarker for liver cirrhosis [14, 16]. Beyond limonene, many other terpenes/terpenoids have been implicated as useful biomarkers, including α-pinene for natural human malarial infection [46]. Other VOCs in Table 1, which are potentially relevant in human health monitoring, include indole, which has been shown to be a biomarker for blood glucose in diabetic patients [47]. Figure 6 shows a hierarchical heatmap of this VOC data, which is autoscaled according to the day of analysis. It should be noted that all volunteer samples were grouped together by a sampling technique to observe methodological differences and not biological variation between volunteers. Within the heatmap, the VOCs are listed as rows and samples are noted in different columns. Red denotes a relatively high VOC signal, green signifies low signal, and black is designated for median values. In addition, a dendrogram on the left-hand side of the heatmap shows how molecular features cluster based on similarities in expression among all collected samples. The heatmap shows that not only are the 15 VOCs significantly enriched in at least one of the breath methods relative to background, but it also displays differences in method sensitivity. For example, the majority of analytes (with the exception of indole) displayed higher GC–MS signals when collected using Tedlar bags.

**Fig. 7:**
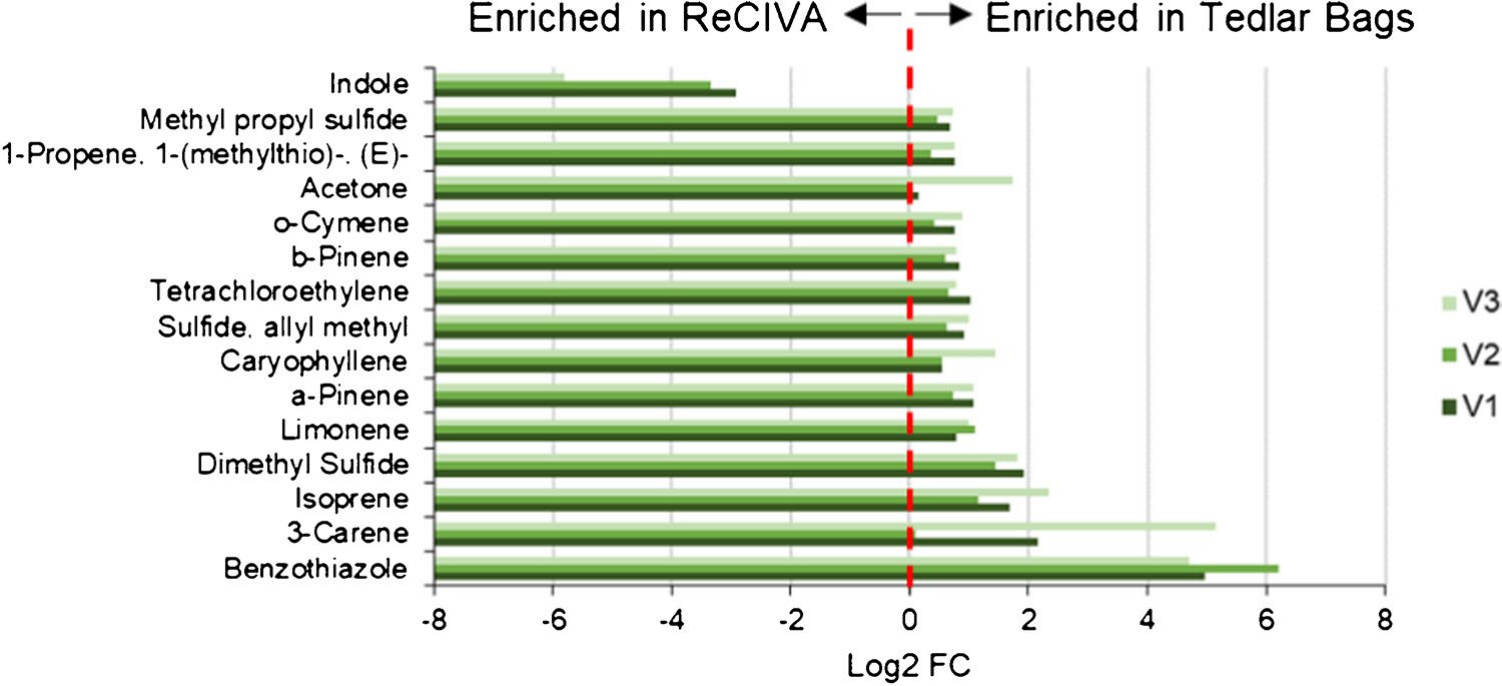
Log_2_ fold change (FC) values for all 15 VOCs across three volunteers, showing the majority of analytes are upregulated in Tedlar bags

All VOCs across all three volunteers, except for acetone, showed statistically significant differences between the two methods (median *p*-value = 9.8 × 10^–6^). To supplement this analysis and determine the degree of enrichment, log_2_ FC values for each volunteer were computed, where positive values correspond to upregulation in Tedlar bags, and negative denotes upregulation in ReCIVA (Fig. 7). For reference, a dotted line is placed at log_2_ FC equal to “0,” and corresponding labels are used to denote which VOCs were more sensitively detected in each method. Indole was the only VOC in which the ReCIVA produced a more sensitive analysis, as across all three volunteers the average log_2_ FC was − 4.03. All other VOCs demonstrated positive log_2_ FC values, with most compounds ranging between 0.6 and 1.0. Benzothiazole was upregulated in Tedlar bags with the highest FC, which makes sense given it was sparsely detected using the ReCIVA. Appendix Table A2 shows VOC ranges for each volunteer regarding both methods, and Appendix Table A3 lists summary statistics (including *p*-values and log_2_ FC values) for the ReCIVA and Tedlar bag comparison. There is no robust scientific rationale for why specific VOCs were upregulated in either device, as there were no distinguishable trends regarding molecular weight, RT, or functional group. However, there are fundamental differences in sampling between the two methods which may or may not account for this variation. For example, as breath is collected using a Tedlar bag, exhaled water vapor condenses on the surface and therefore is not transferred to adsorption tubes. When exhaling directly into the ReCIVA, water vapor instead is likely to condense directly inside the adsorption tube and therefore could impact VOC sensitivity. Another factor that may introduce disparity between the methods is the fact that the inlet diameter of the Tedlar bag is much smaller compared to the ReCIVA. Therefore, it is unknown if this decrease in diameter causes significant resistance when providing a breath sample. Moreover, resistance during breath sampling can have profound effects on the results [48, 49], and this needs to be further studied using manometers.

There are also other considerations when interpreting the results presented. For example, because the ReCIVA uses the CASPER air supply, which provides a clean line of gas for the patient to inhale and subsequently exhale, breath VOC concentrations can be diluted in an unavoidable fashion. It can be noted that a limitation of this study is that the impact of different exhalation volumes on method performance was not evaluated for the ReCIVA. More experimentation is needed to understand the effect of dilution factors, with future research focused on studying VOC sensitivity in the context of exhaled breath sampling volume using this device. It should also be noted that the Tedlar bag breath sampling method used an inline biofilter, which may suppress VOC signal or lead to the generation of new chemical species. As exhaled water vapor and carbon dioxide pass through the filter into the lines of tubing, carbon acid is formed and may be able to interact with on-breath VOCs, depending on the pKa of the target analyte. For example, indole is a biogenic amine that may undergo chemical reactions with the acidic breath condensate solution and therefore may not be detected when breath is sampled using Tedlar bags. Although this factor was not considered, future research should focus on determining the optimal filter to minimize VOC interferences and any chemical reactions.

### Benchmarking method reproducibility

**Fig. 8:**
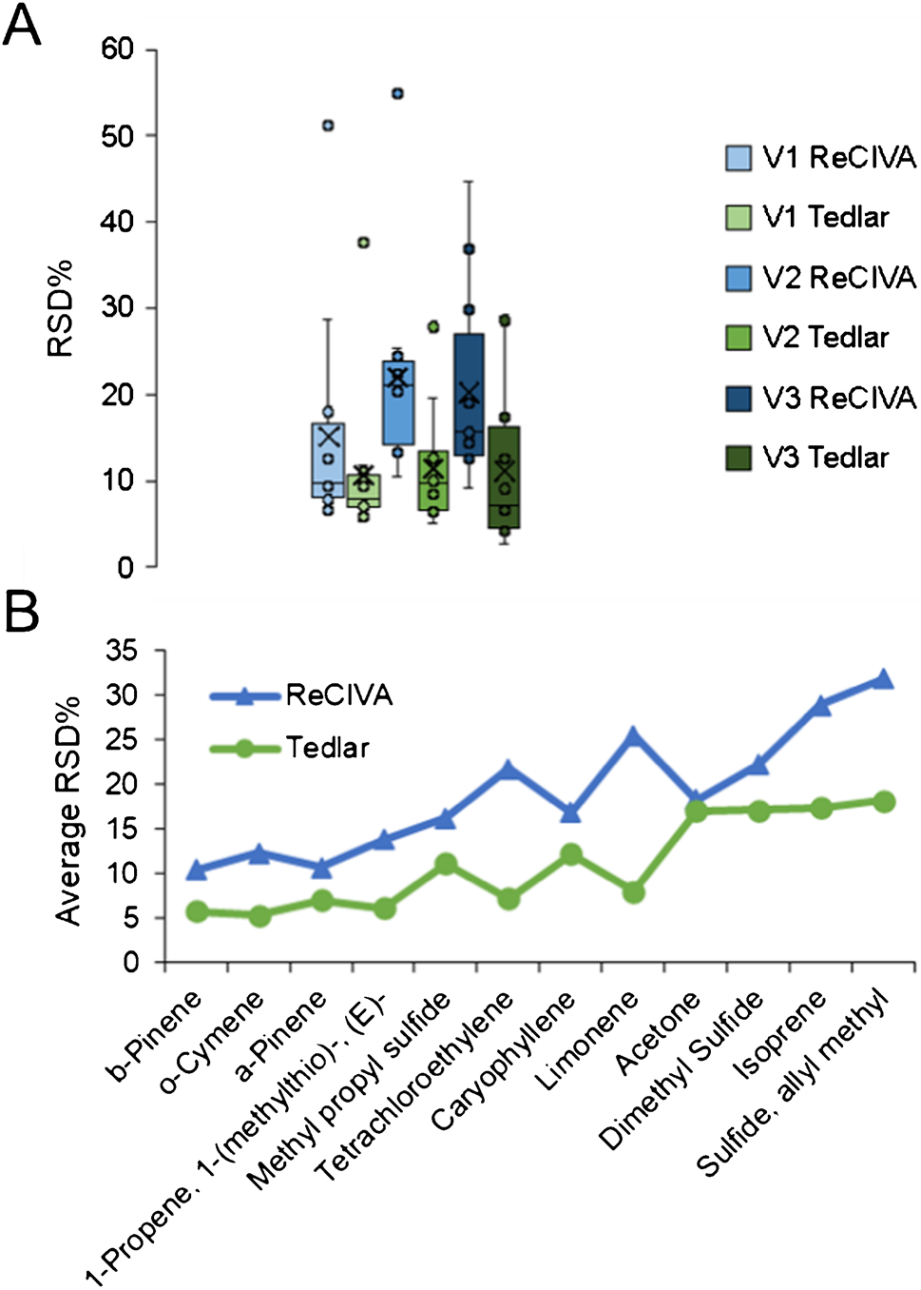
**A** Box/whisker plots for each method and volunteer regarding 12 VOCs for which RSD could be calculated. **B** Scatter plots show the average RSD for both methods across all three volunteers. Taken as a whole, they show that breath-based VOC analysis is significantly more reproducible using Tedlar bags

Reproducibility of breath VOC signals in each method was assessed through calculating and visualizing RSD values across different volunteers. It should be noted that three VOCs were excluded from this analysis (3-carene, benzothiazole, and indole) since they had incomputable RSD values (presence of zero values for a given feature) for at least one of the volunteers in at least one of the methods. First, RSD values were compiled for each volunteer/method and visualized using a box/whisker plot (Fig. 8(A)). For all three volunteers, RSD values from analyses using the ReCIVA were statistically significantly higher relative to Tedlar bags (*p-*value < 0.03 for each of the three volunteers). These results indicate that for this set of 12 VOCs, quantitative analysis of breath collected using a Tedlar bag is significantly more reproducible than using the ReCIVA. Although the box/whisker plot shows this significant difference, it does not illustrate how individual VOCs vary between the methods. Therefore, the average RSD was calculated across different volunteers for each VOC in the two different methods, and these results are displayed in Fig. 8(B). Other than acetone, which had equivocal reproducibility across the two methods, all other breath-based compounds displayed lower RSD values in Tedlar bags. All VOCs within Tedlar bag samples had an average RSD < 20%, where there were many analytes within the ReCIVA data that exceeded this range. For example, isoprene, allyl methyl sulfide, and limonene had an average RSD > 25% when analyzed via the ReCIVA. Although analysis was undertaken in just a few volunteers, the results converged regarding this limited set of VOCs.

### Exploratory multivariate statistical approaches

**Fig. 9:**
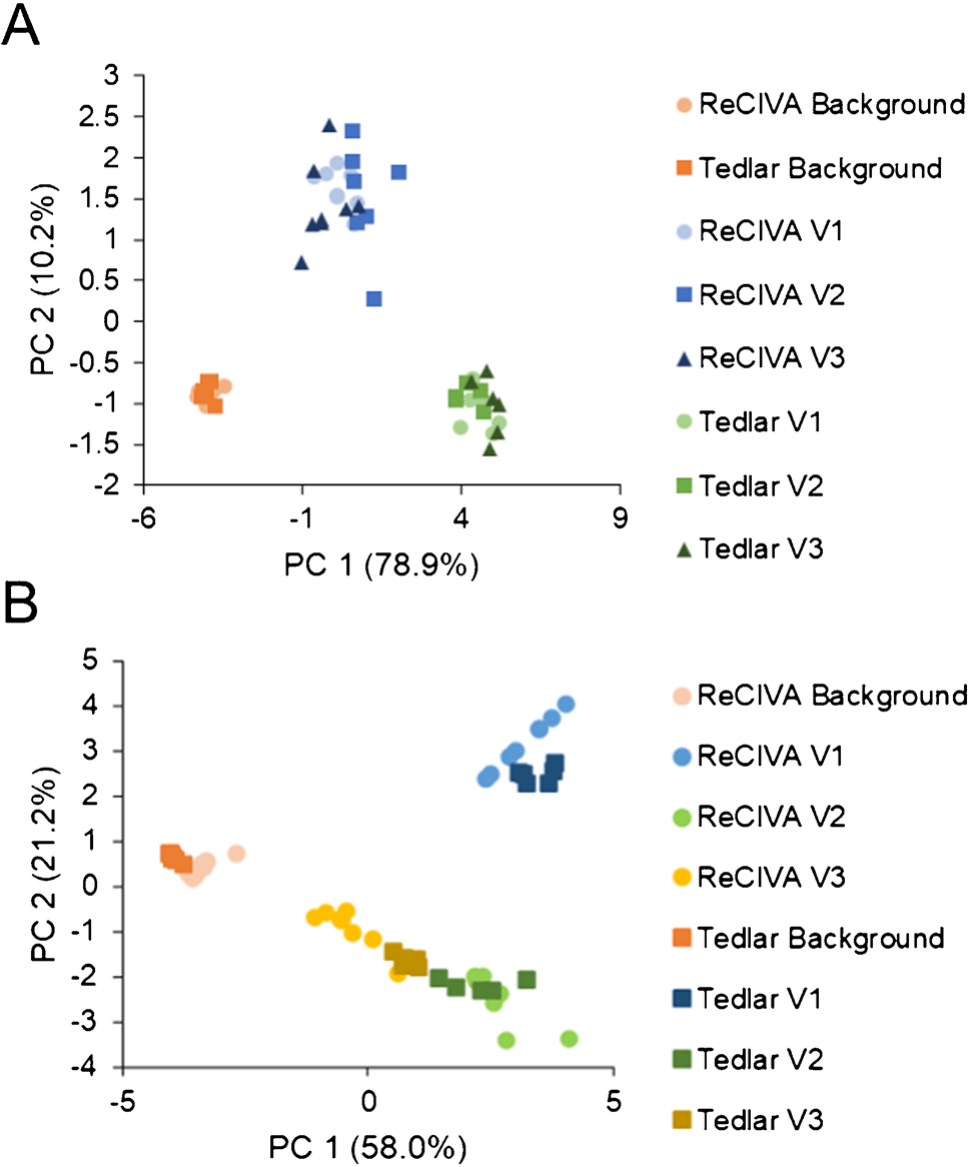
Principal component analyses utilizing data from 15 VOCs which was autoscaled by **A** day of analysis to visualize methodological differences and **B** breath sampling method to determine concordance/agreement

Unsupervised multivariate analysis in the form of PCA was implemented on the set of 15 VOCs to reduce data dimensionality and understand how performance holistically differs due to breath sampling methodology. This analysis is considered to be strictly exploratory, given the limited number of samples in the dataset. First, the VOC data was z-scored according to the day of instrumental analysis (by volunteer). In this way, any variation between the different volunteers is negated and analysis is focused on differences in methodology. Figure 9(A) shows the PCA plot for this analysis in a two-dimensional space, and the first two principal components accounted for approximately 89% of variation within the data. In this plot, different colors correspond to the different methods, and different shapes are utilized consistently for the three volunteers. All breath samples, regardless of the sampling method, were clearly distinguishable from background air samples. Furthermore, ReCIVA and Tedlar bag breath samples could also be stratified with 100% accuracy from each other, but volunteers themselves could not be separated based on sample replicates. After using PCA to probe method-based differences, the data was next autoscaled using a slightly different procedure to focus on variation between the different volunteers and not the methods themselves. In this way, it can be assessed if the VOC data collected using the two different methods are reflective of one another. Here, VOC signals were z-scored according to method of analysis, and PCA was implemented on the normalized dataset (Fig. 9(B)). In the PCA plot, different colors correspond to different volunteers, and the two methods are denoted using two unique shapes. The first two principal components nearly accounted for 80% of the variation within the data, and it can be observed there is a high degree of agreement between the ReCIVA and Tedlar bag methods. For example, there was a clear overlap between the Tedlar bag/ReCIVA background samples, and volunteers showed homologous PCA scores regardless of breath sampling methodology. Moreover, the PCA results from both breath sampling methods converged. Although all three volunteers could be distinguished, the exhaled VOC profiles of volunteers 2 and 3 are relatively similar and to a greater extent more distinguishable from volunteer 1. This result was seen in both Tedlar bag and ReCIVA breath sampling methodology.

### Analysis of method agreement

**Fig. 10:**
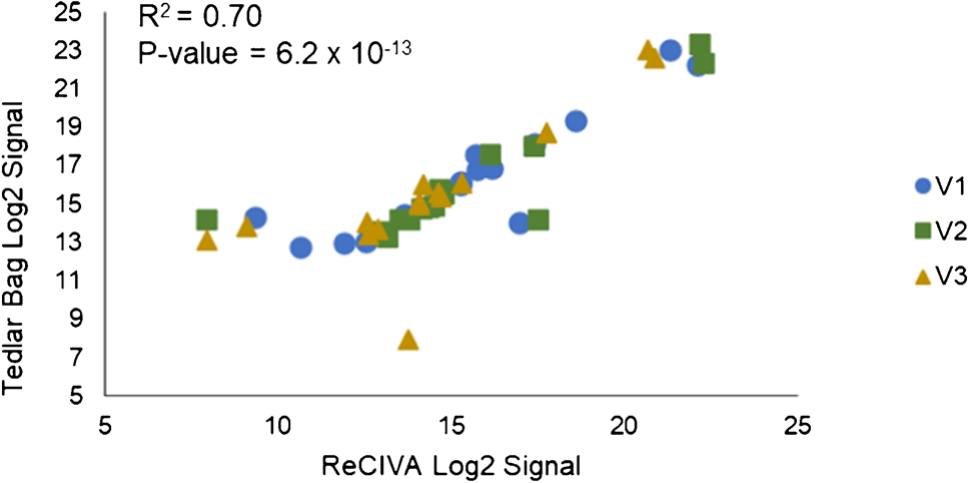
Scatter plot showing a statistically significant correlation in average log_2_ VOC signals between ReCIVA and Tedlar bags across all three volunteers

To further explore degrees of method agreement, the average signal for each of the 15 VOCs was calculated for each method in all three volunteers. Because the two methods had slightly varying numbers of replicates, a direct correlation of repeated measures could not be conducted. Integrated signals underwent log_2_ transformation, and a scatter plot showing the correlation between Tedlar bag and ReCIVA VOC signals is shown in Fig. 10. Here, different shapes/colors correspond to the three volunteers, and there is a statistically significant correlation in VOC analysis between ReCIVA and Tedlar bags (*R*^2^ = 0.70, *p*-value = 6.2 × 10^–13^). This indicates that VOC profiles stay consistent regardless of the breath sampling methodology which is employed. Inadvertently, this may suggest that any resistance in airway sampling induced by Tedlar bags is negligible and/or does not affect VOC results. This is supported by previous studies, which have indicated that varying exhalation rate into the ReCIVA does not vary results [37]. The data also suggest that similar breath-based results can be achieved using either method. Although there is a high degree of agreement between the methods, this does not inherently indicate that results are translatable between devices. Given that Tedlar bags in general produced higher VOC signals in this study, these signals would need to be normalized for ultimate alignment and translatability.

Taken as a whole, breath analysis is viable in Tedlar bags or ReCIVA, but in general, Tedlar bags are more sensitive and reproducible. A limitation to the study is that true quantitative concentration levels, along with values for limits of detection/quantification (LOD/LOQ) were not elucidated. This is because currently, there is no gold standard method for benchmarking method performance or quantitating VOC levels in breath using offline techniques. Most studies undertake a pseudo-quantitative approach for biostatistical approaches, where normalized GC–MS signals are available for analysis. Given this significant downfall in literature, the current study presents a new approach for validating breath-based results through collection using two different breath sampling methods and drawing correlations between them. Until methods are established for quantitation and determining LOD/LOQ values, the researchers suggest using the ReCIVA and bag-based sampling in parallel for results verification. Beyond the current study, there have been different initiatives such as the “peppermint experiment” which has been adopted by an international consortium of breath research teams to provide a method for benchmarking different sampling procedures [50, 51]. This method seeks to validate breath sampling methodology to observe the washout time of observed peppermint-associated volatiles. It is anticipated that in the future, repeated/sequential breath sampling coupled with peppermint experiments will pave the way for cross-validating platform performance.

## Conclusion

The current study makes strides toward normalizing and standardizing methods for offline breath sampling, through exploring various qualitative and quantitative factors that influence VOC analysis when utilizing Tedlar bags for exhaled breath collection. Important lessons learned here include the requirements for PFA or PTFE tubing in breath collection/transfer and the importance of breath fractionation in bolstering sensitivity/reproducibility. After parameter optimization, the performance of two breath collection platforms (Tedlar bags as well as the ReCIVA) was benchmarked and compared in a quantitative fashion through a robust series of experiments. Through analyzing numerous consecutive breath sample replicates from three different healthy volunteers, it was demonstrated that analysis of VOCs in Tedlar bags not only resulted in higher VOC sensitivity, but also significantly superior reproducibility. It should be noted that the ReCIVA did show higher performance with regard to the detection of indole, a potentially important biomarker implicated for a wide range of medical conditions [47, 52]. Although more sensitive and reproducible using Tedlar bags, univariate and multivariate chemometric analyses revealed that the results from both instruments converged, and therefore both platforms may be used for VOC biomarker discovery in exhaled breath. It should be noted that these results were obtained with a low number of volunteers, and therefore the results are preliminary. More experiments are needed to draw more robust conclusions on device performance and ability to discern true biological variability. The team is currently working to expand these results to cohorts of patients diagnosed with cystic fibrosis, to increase sample numbers that are acceptable to industry standards and demonstrate clinical utility. Further work is warranted regarding offline breath sampling and the exploration/standardization of other factors, including those related to exhaled breath sampling resistance and respiratory physiology. It is of critical importance to develop optimized and standardized methods for sampling that can be widely utilized by the breath research community, which in turn will facilitate the production of more reliable data that can be cross-referenced and leveraged by different research groups. Ultimately, this will permit larger studies for proper biomarker validation and therefore can speed up the acceptance and translation of breath-based assays in clinical applications.

## Supplementary Information

Below is the link to the electronic supplementary material.

Supplementary file1 (DOCX 37 KB)

## Data Availability

The authors provide no restriction on the availability of methods, protocols, instrumentation, and data utilized in the following article. All data will be available from the corresponding author through reasonable requests.

## References

[1] Boots AW, van Berkel JJ, Dallinga JW, Smolinska A, Wouters EF, van Schooten FJ. The versatile use of exhaled volatile organic compounds in human health and disease. J Breath Res. 2012;6(2):027108. 22621865 10.1088/1752-7155/6/2/027108

[2] Haworth JJ, Pitcher CK, Ferrandino G, Hobson AR, Pappan KL, Lawson JLD. Breathing new life into clinical testing and diagnostics: perspectives on volatile biomarkers from breath. Crit Rev Clin Lab Sci. 2022;59(5):353–72. 35188863 10.1080/10408363.2022.2038075

[3] Moura PC, Raposo M, Vassilenko V. Breath volatile organic compounds (VOCs) as biomarkers for the diagnosis of pathological conditions: a review. Biomedical Journal. 2023;46(4):100623. 37336362 10.1016/j.bj.2023.100623 PMC10339195

[4] Janfaza S, Khorsand B, Nikkhah M, Zahiri J. Digging deeper into volatile organic compounds associated with cancer. Biol Methods Protocol. 2019;4(1):bpz014. 10.1093/biomethods/bpz014 PMC6994028 32161807

[5] Bos LDJ, Sterk PJ, Schultz MJ. Volatile metabolites of pathogens: a systematic review. PLoS Pathog. 2013;9(5):e1003311. 23675295 10.1371/journal.ppat.1003311 PMC3649982

[6] Hancock G, Sharma S, Galpin M, Lunn D, Megson C, Peverall R, Richmond G, Ritchie GAD, Owen KR. The correlation between breath acetone and blood betahydroxybutyrate in individuals with type 1 diabetes. J Breath Res. 2021;15(1):017101. 10.1088/1752-7163/abbf37 33027776

[7] Saasa V, Beukes M, Lemmer Y, Mwakikunga B. Blood ketone bodies and breath acetone analysis and their correlations in type 2 diabetes mellitus. Diagn. 2019;9(4):224. 10.3390/diagnostics9040224 PMC6963753 31861135

[8] Wang P, Huang Q, Meng S, Mu T, Liu Z, He M, Li Q, Zhao S, Wang S, Qiu M. Identification of lung cancer breath biomarkers based on perioperative breathomics testing: a prospective observational study. eClinicalMedicine. 2022;47:101384. 10.1016/j.eclinm.2022.101384 PMC9035731 35480076

[9] Phillips M, Gleeson K, Hughes JMB, Greenberg J, Cataneo RN, Baker L, McVay WP. Volatile organic compounds in breath as markers of lung cancer: a cross-sectional study. The Lancet. 1999;353(9168):1930–3. 10.1016/S0140-6736(98)07552-7 10371572

[10] Yang H-Y, Wang Y-C, Peng H-Y, Huang C-H. Breath biopsy of breast cancer using sensor array signals and machine learning analysis. Sci Rep. 2021;11(1):103. 33420275 10.1038/s41598-020-80570-0 PMC7794369

[11] Phillips M, Cataneo RN, Saunders C, Hope P, Schmitt P, Wai J. Volatile biomarkers in the breath of women with breast cancer. J Breath Res. 2010;4(2):026003. 21383471 10.1088/1752-7155/4/2/026003

[12] Ren Y, Wang F, Zhu Z, Luo R, Lv G, Cui H. Breath biomarkers for esophageal cancer: identification, quantification, and diagnostic modeling. Anal Sci. 2025;41:965–76. 40232623 10.1007/s44211-025-00769-x

[13] Chou H, Godbeer L, Allsworth M, Boyle B, Ball ML. Progress and challenges of developing volatile metabolites from exhaled breath as a biomarker platform. Metabolomics. 2024;20(4):72. 38977623 10.1007/s11306-024-02142-x PMC11230972

[14] Patnaik RK, Lin Y-C, Agarwal A, Ho M-C, Yeh JA. A pilot study for the prediction of liver function related scores using breath biomarkers and machine learning. Sci Rep. 2022;12(1):2032. 35132067 10.1038/s41598-022-05808-5 PMC8821604

[15] Thomas JN, Roopkumar J, Patel T. Machine learning analysis of volatolomic profiles in breath can identify non-invasive biomarkers of liver disease: a pilot study. PLoS ONE. 2021;16(11):e0260098. 34847181 10.1371/journal.pone.0260098 PMC8631657

[16] O’Hara ME, Fernández Del Río R, Holt A, Pemberton P, Shah T, Whitehouse T, Mayhew CA. Limonene in exhaled breath is elevated in hepatic encephalopathy. J Breath Res. 2016;10(4):046010. 27869108 10.1088/1752-7155/10/4/046010 PMC5500822

[17] Pijls KE, Smolinska A, Jonkers DMAE, Dallinga JW, Masclee AAM, Koek GH, van Schooten F-J. A profile of volatile organic compounds in exhaled air as a potential non-invasive biomarker for liver cirrhosis. Sci Rep. 2016;6(1):19903. 26822454 10.1038/srep19903 PMC4731784

[18] Belizário JE, Faintuch J, Malpartida MG. Breath biopsy and discovery of exclusive volatile organic compounds for diagnosis of infectious diseases. Front Cell Infect Microbiol. 2021;10:564194–564194. 33520731 10.3389/fcimb.2020.564194 PMC7839533

[19] Ghosh C, Leon A, Koshy S, Aloum O, Al-Jabawi Y, Ismail N, Weiss ZF, Koo S. Breath-based diagnosis of infectious diseases: a review of the current landscape. Clin Lab Med. 2021;41(2):185–202. 34020759 10.1016/j.cll.2021.03.002 PMC8141093

[20] Bajo-Fernández M, Souza-Silva ÉA, Barbas C, Rey-Stolle MF, García A. GC-MS-based metabolomics of volatile organic compounds in exhaled breath: applications in health and disease. A Review Front Mol Biosci. 2024;10:1295955. 10.3389/fmolb.2023.1295955 PMC10828970 38298553

[21] Ge D, Zhou J, Chu Y, Lu Y, Zou X, Xia L, Liu Y, Huang C, Shen C, Zhang L, Wang H, Chu Y. Distinguish oral-source VOCs and control their potential impact on breath biomarkers. Anal Bioanal Chem. 2022;414(6):2275–84. 34982180 10.1007/s00216-021-03866-8

[22] Lu Y, Niu W, Zou X, Shen C, Xia L, Huang C, Wang H, Jiang H, Chu Y. Glass bottle sampling solid phase microextraction gas chromatography mass spectrometry for breath analysis of drug metabolites. J Chromatogr A. 2017;1496:20–4. 28365077 10.1016/j.chroma.2017.03.061

[23] Lecharlier A, Bouyssiere B, Carrier H, Hécho IL. Promises of a new versatile field-deployable sorbent tube thermodesorber by application to BTEX analysis in CH4. Talanta Open. 2021;4:100066.

[24] He Y, Su Z, Sha T, Yu X, Guo H, Tao Y, Liao L, Zhang Y, Lu G, Lu G, Gong W. Collection methods of exhaled volatile organic compounds for lung cancer screening and diagnosis: a systematic review. J Thorac Dis. 2024;16(11):7978–98. 39678905 10.21037/jtd-24-1001 PMC11635225

[25] Miekisch W, Kischkel S, Sawacki A, Liebau T, Mieth M, Schubert JK. Impact of sampling procedures on the results of breath analysis. J Breath Res. 2008;2(2):026007. 21383448 10.1088/1752-7155/2/2/026007

[26] Španěl P, Dryahina K, Smith D. A quantitative study of the influence of inhaled compounds on their concentrations in exhaled breath. J Breath Res. 2013;7(1):017106. 23445832 10.1088/1752-7155/7/1/017106

[27] Amann A, Miekisch W, Pleil J, Risby T, Schubert J. Methodological issues of sample collection and analysis of exhaled breath. European Respiratory Monograph 2010; p. 96–114.

[28] Lourenço C, Turner C. Breath analysis in disease diagnosis: methodological considerations and applications. 2014;4(2):465–98. 10.3390/metabo4020465 PMC4101517 24957037

[29] Schulz E, Woollam M, Grocki P, Davis MD, Agarwal M. Methods to detect volatile organic compounds for breath biopsy using solid-phase microextraction and gas chromatography-mass spectrometry. Molecules. 2023;28(11):4533. 37299010 10.3390/molecules28114533 PMC10254745

[30] Westphal K, Dudzik D, Waszczuk-Jankowska M, Graff B, Narkiewicz K, Markuszewski MJ. Common strategies and factors affecting off-line breath sampling and volatile organic compounds analysis using thermal desorption-gas chromatography-mass spectrometry (TD-GC-MS). Metabolites. 2023;13(1):8. 10.3390/metabo13010008 PMC9866406 36676933

[31] Czippelová B, Nováková S, Šarlinová M, Baranovičová E, Urbanová A, Turianiková Z, Krohová J, Halašová E, Škovierová H. Impact of breath sample collection method and length of storage of breath samples in Tedlar bags on the level of selected volatiles assessed using gas chromatography-ion mobility spectrometry (GC-IMS). J Breath Res. 2024;18(3):036004. 10.1088/1752-7163/ad4736 38701772

[32] Bhavra K, Wilde M, Richardson M, Cordell R, Thomas P, Zhao B, Bryant L, Brightling C, Ibrahim W, Salman D, Siddiqui S, Monks P, Gaillard E. Paediatric breath VOC analysis using the ReCIVA breath sampler and batch effects. Eur Respir J. 2022;60(suppl 66):2761. 10.1088/1752-7163/ac5526 35168217

[33] Arulvasan W, Chou H, Greenwood J, Ball ML, Birch O, Coplowe S, Gordon P, Ratiu A, Lam E, Hatch A, Szkatulska M, Levett S, Mead E, Charlton-Peel C, Nicholson-Scott L, Swann S, van Schooten F-J, Boyle B, Allsworth M. High-quality identification of volatile organic compounds (VOCs) originating from breath. Metabolomics. 2024;20(5):102. 39242444 10.1007/s11306-024-02163-6 PMC11379754

[34] Chen J, Ji Y, Liu Y, Cen Z, Chen Y, Zhang Y, Li X, Li X. Exhaled volatolomics profiling facilitates personalized screening for gastric cancer. Cancer Lett. 2024;590:216881. 38614384 10.1016/j.canlet.2024.216881

[35] Markar SR, Brodie B, Chin S-T, Romano A, Spalding D, Hanna GB. Profile of exhaled-breath volatile organic compounds to diagnose pancreatic cancer. Br J Surg. 2018;105(11):1493–500. 30019405 10.1002/bjs.10909

[36] Altomare DF, Picciariello A, Rotelli MT, De Fazio M, Aresta A, Zambonin CG, Vincenti L, Trerotoli P, De Vietro N. Chemical signature of colorectal cancer: case–control study for profiling the breath print. BJS Open. 2020;4(6):1189–99. 32990407 10.1002/bjs5.50354 PMC8444279

[37] Harshman SW, Pitsch RL, Davidson CN, Lee EM, Scott AM, Hill EM, Mainali P, Brooks ZE, Strayer KE, Schaeublin NM, Wiens TL, Brothers MC, Drummond LA, Yamamoto DP, Martin JA. Evaluation of a standardized collection device for exhaled breath sampling onto thermal desorption tubes. J Breath Res. 2020;14(3):036004. 32155613 10.1088/1752-7163/ab7e3b

[38] Di Gilio A, Palmisani J, Ventrella G, Facchini L, Catino A, Varesano N, Pizzutilo P, Galetta D, Borelli M, Barbieri P, Licen S, de Gennaro G. Breath Analysis: Comparison among Methodological Approaches for Breath Sampling. 2020;25(24):5823. 10.3390/molecules25245823 PMC7763204 33321824

[39] Woollam M, Siegel A, Grocki P, Saunders JL, Sanders DB, Agarwal M, Davis MD. Preliminary method for profiling volatile organic compounds in breath that correlate with pulmonary function and other clinical traits of subjects diagnosed with cystic fibrosis: a pilot study. J Breath Res. 2022;16(2):027103. 10.1088/1752-7163/ac522f 35120338

[40] Woollam M, Angarita-Rivera P, Siegel AP, Kalra V, Kapoor R, Agarwal M. Exhaled VOCs can discriminate subjects with COVID-19 from healthy controls. J Breath Res. 2022;16(3):036002. 10.1088/1752-7163/ac696a 35453137

[41] Siegel AP, Daneshkhah A, Hardin DS, Shrestha S, Varahramyan K, Agarwal M. Analyzing breath samples of hypoglycemic events in type 1 diabetes patients: towards developing an alternative to diabetes alert dogs. J Breath Res. 2017;11(2):026007. 28569238 10.1088/1752-7163/aa6ac6

[42] Schulz E, Woollam M, Vashistha S, Agarwal M. Quantifying exhaled acetone and isoprene through solid phase microextraction and gas chromatography-mass spectrometry. Anal Chim Acta. 2024;1301:342468. 38553125 10.1016/j.aca.2024.342468

[43] Beauchamp J, Herbig J, Gutmann R, Hansel A. On the use of Tedlar® bags for breath-gas sampling and analysis. J Breath Res. 2008;2(4):046001. 21386188 10.1088/1752-7155/2/4/046001

[44] Koziel JA, Spinhirne JP, Lloyd JD, Parker DB, Wright DW, Kuhrt FW. Evaluation of sample recovery of malodorous livestock gases from air sampling bags, solid-phase microextraction fibers, Tenax TA sorbent tubes, and sampling canisters. J Air Waste Manag Assoc. 2005;55(8):1147–57. 16187584 10.1080/10473289.2005.10464711

[45] Akdeniz N, Janni KA, Jacobson LD, Hetchler BP. Comparison of gas sampling bags to temporarily store hydrogen sulfide, ammonia, and greenhouse gases. Transact ASABE. 2011;54(2):653–61.

[46] Schaber CL, Katta N, Bollinger LB, Mwale M, Mlotha-Mitole R, Trehan I, Raman B, Odom John AR. Breathprinting reveals malaria-associated biomarkers and mosquito attractants. J Infect Dis. 2018;217(10):1553–60. 29415208 10.1093/infdis/jiy072 PMC6279169

[47] Fink H, Maihöfer T, Bender J, Schulat J. Indole as a new tentative marker in exhaled breath for non-invasive blood glucose monitoring of diabetic subjects. J Breath Res. 2022;16(2):026001. 10.1088/1752-7163/ac4610 34942609

[48] Sukul P, Schubert JK, Oertel P, Kamysek S, Taunk K, Trefz P, Miekisch W. FEV manoeuvre induced changes in breath VOC compositions: an unconventional view on lung function tests. Sci Rep. 2016;6(1):28029. 27311826 10.1038/srep28029 PMC4911606

[49] Sukul P, Schubert JK, Kamysek S, Trefz P, Miekisch W. Applied upper-airway resistance instantly affects breath components: a unique insight into pulmonary medicine. J Breath Res. 2017;11(4):047108. 28925377 10.1088/1752-7163/aa8d86

[50] Henderson B, Ruszkiewicz DM, Wilkinson M, Beauchamp JD, Cristescu SM, Fowler SJ, Salman D, Francesco FD, Koppen G, Langejürgen J, Holz O, Hadjithekli A, Moreno S, Pedrotti M, Sinues P, Slingers G, Wilde M, Lomonaco T, Zanella D, Zenobi R, Focant J-F, Grassin-Delyle S, Franchina FA, Malásková M, Stefanuto P-H, Pugliese G, Mayhew C, Thomas CLP. A benchmarking protocol for breath analysis: the peppermint experiment. J Breath Res. 2020;14(4):046008. 32604084 10.1088/1752-7163/aba130

[51] Wilkinson M, White I, Hamshere K, Holz O, Schuchardt S, Bellagambi FG, Lomonaco T, Biagini D, Di FF, Fowler SJ. On Behalf of the Peppermint, I., The peppermint breath test: a benchmarking protocol for breath sampling and analysis using GC–MS. J Breath Res. 2021;15(2):026006. 10.1088/1752-7163/abd28c 33302258

[52] Dadamio J, Van den Velde S, Laleman W, Van Hee P, Coucke W, Nevens F, Quirynen M. Breath biomarkers of liver cirrhosis. J Chromatogr B. 2012;905:17–22. 10.1016/j.jchromb.2012.07.025 22921634

