# Supplementary material for: Systematic comparison of methods for offline breath sampling
Source: Anal Bioanal Chem. 2025 Aug 5;417(22):5061–76. doi: 10.1007/s00216-025-06025-5 (PMC12402010; doi:10.1007/s00216-025-06025-5)
Supplement: Supplementary file 1 — Supplementary file1 (DOCX 37 KB) [file 216_2025_6025_MOESM1_ESM.docx]

**APPENDIX A: EXPERIMENTAL INFORMATION AND ADDITIONAL RESULTS**

**Systematic Comparison of Methods for Offline Breath Sampling**

*Mark Woollam^a,b*^, Andrew Christensen^b^, Eray Schulz^a,b^, Serenidy Eckerle^b^, Michael D. Davis^c,d^, Don B. Sanders^c^, and Mangilal Agarwal^a,b,e*^*

^a^Department of Chemistry & Chemical Biology, Indiana University Indianapolis, IN, 46202

^b^Integrated Nanosystems Development Institute, Indiana University Indianapolis, IN, 46202

^c^Division of Pulmonology, Allergy, and Sleep Medicine Riley Hospital for Children at Indiana University School of Medicine, Indianapolis, IN, 46202

^d^Wells Center for Pediatric Research, Riley Hospital for Children at Indiana University School of Medicine, Indianapolis, IN, 46202

^e^Department of Biomedical Engineering & Informatics, Indiana University Indianapolis, IN, 46202

^*^Corresponding authors: [mwoollam@iu.edu](mailto:mwoollam@iu.edu) & [agarwal@iu.edu](mailto:agarwal@iu.edu), 317-278-9792

Table A1. Overview of all parameters and samples collected for each of the experiments presented for bag-based optimization and platform validation.

| **Bag-based Optimization Experiment 1a** | |  |  |  |  |  |
| --- | --- | --- | --- | --- | --- | --- |
| Tubing Type | Sampling Device | Sample Type | Volume (mL) | Flow Rate (mL/min) | Fractionation | Replicates |
| *PFA Tubing* | Tedlar Bags | UHP Nitrogen | 500 | 250 | NA | 3 |
| PTFE Tubing | Tedlar Bags | UHP Nitrogen | 500 | 250 | NA | 3 |
| Tygon Tubing | Tedlar Bags | UHP Nitrogen | 500 | 250 | NA | 3 |
| **Bag-based Optimization Experiment 1b** | |  |  |  |  |  |
| Tubing Type | Sampling Device | Sample Type | Volume (mL) | Flow Rate (mL/min) | Fractionation | Replicates |
| *PFA Tubing* | Tedlar Bags | Exhaled Breath | 500 | 250 | No | 3 (1 volunteer) |
| PTFE Tubing | Tedlar Bags | Exhaled Breath | 500 | 250 | No | 3 (1 volunteer) |
| Tygon Tubing | Tedlar Bags | Exhaled Breath | 500 | 250 | No | 3 (1 volunteer) |
| **Bag-based Optimization Experiment 2** | |  |  |  |  |  |
| Sampling Device | Sample Type | Volume (mL) | Flow Rate (mL/min) | Fractionation | Replicates |  |
| *Tedlar Bags* | UHP Nitrogen | 500 | 250 | NA | 3 |  |
| Multi-Layer Foil Bags | UHP Nitrogen | 500 | 250 | NA | 3 |  |
| **Bag-based Optimization Experiment 3** | |  |  |  |  |  |
| Fractionation | Sampling Device | Sample Type | Volume (mL) | Flow Rate (mL/min) | Replicates |  |
| *Yes* | Tedlar Bags | Exhaled Breath | 500 | 250 | 3 (1 volunteer) |  |
| No | Tedlar Bags | Exhaled Breath | 500 | 250 | 3 (1 volunteer) |  |
| **Bag-based Optimization Experiment 4a** | |  |  |  |  |  |
| Sample Type | Volume (mL) | Flow Rate (mL/min) | Replicates |  |  |  |
| Exhaled Breath | 500 | 250 | 3 (1 volunteer) |  |  |  |
| Exhaled Breath | 1000 | 250 | 3 (1 volunteer) |  |  |  |
| Exhaled Breath | 1500 | 250 | 3 (1 volunteer) |  |  |  |
| **Bag-based Optimization Experiment 4b** | |  |  |  |  |  |
| Sample Type | Volume (mL) | Flow Rate (mL/min) | Replicates |  |  |  |
| Exhaled Breath | 1500 | 250 | 3 (1 volunteer) |  |  |  |
| Exhaled Breath | *2000* | 250 | 3 (1 volunteer) |  |  |  |
| Exhaled Breath | 2500 | 250 | 3 (1 volunteer) |  |  |  |
| **Bag-based Optimization Experiment 5** | |  |  |  |  |  |
| Sample Type | Volume (mL) | Flow Rate (mL/min) | Replicates |  |  |  |
| Exhaled Breath | 2000 | 125 | 3 (1 volunteer) |  |  |  |
| Exhaled Breath | 2000 | 250 | 3 (1 volunteer) |  |  |  |
| Exhaled Breath | 2000 | 375 | 3 (1 volunteer) |  |  |  |
| Exhaled Breath | 2000 | 500 | 3 (1 volunteer) |  |  |  |
| **Platform Comparison and Benchmarking Experiment** | |  |  |  |  |  |
| Sampling Device | Sample Type | Volume (mL) | Flow Rate (mL/min) | Fractionation | Replicates |  |
| Tedlar Bags | Exhaled Breath | 1250 | 200 | Yes | n > 15 (3 volunteers) |  |
| ReCIVA | Exhaled Breath | 1250 | 200 | Yes | n > 15 (3 volunteers) |  |

Table A2. Ranges for Tedlar bag and ReCIVA sampling devices when comparing on-breath VOC signals across three unique volunteers.

| VOC Name | Tedlar V1min | Tedlar V1max | ReCIVA V1min | ReCIVA V1max | Tedlar V2min | Tedlar V2max | ReCIVA V2min | ReCIVA V2max | Tedlar V3min | Tedlar V3max | ReCIVA V3min | ReCIVA V3max |
| --- | --- | --- | --- | --- | --- | --- | --- | --- | --- | --- | --- | --- |
| Acetone | 4395751 | 5744608 | 3478665 | 5854889 | 4860009 | 6288363 | 3433551 | 6796479 | 4095409 | 9460872.59 | 1630336 | 2437475 |
| Isoprene | 7093666 | 9484784 | 1664352 | 3914159 | 8943104 | 12582514 | 3679606 | 6359975 | 4781861 | 10676398 | 1016102 | 3039770 |
| Dimethyl Sulfide | 169055 | 215212 | 45219 | 66015 | 145047 | 268252 | 48939 | 104978 | 51532 | 73991 | 13660 | 31949 |
| Sulfide, allyl methyl | 17440 | 87923 | 250 | 60715 | 217145 | 310502 | 142306 | 273023 | 404102 | 454774 | 174946 | 277932 |
| Methyl propyl sulfide | 110527 | 133367 | 66555 | 85519 | 19290 | 32798 | 16515 | 29596 | 38999 | 47397 | 19087 | 35247 |
| 1-Propene, 1-(methylthio)- | 267268 | 316270 | 153151 | 192401 | 26100 | 32369 | 18546 | 33359 | 66432 | 73500 | 33237 | 49295 |
| Tetrachloroethylene | 7237 | 8758 | 3551 | 4175 | 17320 | 20270 | 10242 | 15355 | 9984 | 12319 | 4746 | 13091 |
| a-Pinene | 104934 | 120331 | 45701 | 63759 | 40365 | 51449 | 24744 | 34452 | 33601 | 40468 | 15917 | 20441 |
| b-Pinene | 21320 | 25945 | 11603 | 14469 | 25729 | 29131 | 15353 | 21002 | 12362 | 13988 | 6635 | 9419 |
| 3-carene | 6479 | 7847 | 250 | 6747 | 8691 | 10879 | 250 | 45393 | 8328 | 9689 | 250 | 250 |
| o-Cymene | 60103 | 72378 | 34630 | 44476 | 17247 | 20081 | 10854 | 16522 | 31020 | 33192 | 14196 | 21833 |
| Limonene | 612893 | 750336 | 363868 | 430090 | 50471 | 61378 | 4917 | 38516 | 45824 | 58275 | 20342 | 32610 |
| Benzothiazole | 14264 | 25570 | 250 | 3451 | 17993 | 18740 | 250 | 250 | 9272 | 18898 | 250 | 2659 |
| Indole | 10544 | 28842 | 117479 | 140342 | 3337 | 32303 | 138941 | 265783 | 250 | 250 | 8386 | 23199 |
| Caryophyllene | 7411 | 9534 | 4796 | 7340 | 10912 | 13874 | 4765 | 10715 | 11932 | 20867 | 5148 | 7602 |

Table A3. Tedlar bag and ReCIVA statistical comparison results (*p-values* and log_2_FC values) across all three volunteers for the 15 qualified on-breath VOCs.

|  | p-values | | | Log2 FC | | |
| --- | --- | --- | --- | --- | --- | --- |
| VOC Name | V1 | V2 | V3 | V1 | V2 | V3 |
| Acetone | 2.57E-01 | 7.31E-01 | 1.73E-03 | 0.14 | 0.05 | 1.73 |
| Isoprene | 4.23E-08 | 1.36E-04 | 8.36E-04 | 1.69 | 1.14 | 2.36 |
| Dimethyl Sulfide | 6.51E-06 | 5.50E-03 | 1.42E-06 | 1.91 | 1.46 | 1.82 |
| Sulfide, allyl methyl | 3.07E-02 | 2.17E-03 | 1.07E-07 | 0.91 | 0.61 | 0.99 |
| Methyl propyl sulfide | 1.17E-06 | 3.40E-02 | 5.42E-06 | 0.68 | 0.46 | 0.72 |
| 1-Propene, 1-(methylthio)-, (E)- | 1.33E-07 | 1.14E-02 | 4.10E-08 | 0.75 | 0.37 | 0.76 |
| Tetrachloroethylene | 3.97E-06 | 9.10E-06 | 2.13E-03 | 1.03 | 0.66 | 0.79 |
| a-Pinene | 2.13E-09 | 7.56E-05 | 9.66E-08 | 1.06 | 0.72 | 1.08 |
| b-Pinene | 1.66E-06 | 1.99E-06 | 2.00E-08 | 0.84 | 0.59 | 0.79 |
| 3-carene | 4.11E-04 | 9.34E-01 | 1.53E-07 | 2.15 | 0.08 | 5.14 |
| o-Cymene | 4.58E-07 | 6.38E-04 | 2.43E-08 | 0.76 | 0.40 | 0.88 |
| Limonene | 1.04E-05 | 1.19E-03 | 9.10E-07 | 0.79 | 1.09 | 0.99 |
| Benzothiazole | 4.10E-05 | 1.89E-08 | 1.97E-04 | 4.98 | 6.20 | 4.71 |
| Indole | 2.47E-12 | 2.22E-05 | 9.90E-05 | -2.94 | -3.35 | -5.82 |
| Caryophyllene | 6.72E-05 | 1.61E-03 | 1.61E-04 | 0.54 | 0.54 | 1.44 |
